# The Early Diagnosis of Perforation in Typhoid Fever

**Published:** 1904-01-23

**Authors:** 


### THE EARLY DIAGNOSIS OF PERFORATION IN TYPHOID FEVER.

Some disappointment may have been felt that surgery has not achieved more distinct success than has up to the present time been obtained in saving life after perforation of the bowel in the course of enteric fever. This, however, is probably not the fault of the surgeons. As Dr. Hector Mackenzie[1] points out, it is rather to be attributed to delay in resorting to their assistance. He emphasises the importance of every practitioner who has to treat a case of typhoid fever being fully alive to the risk of perforation and keeping a careful watch for those symptoms and signs by the observation of which it is alone possible to arrive at an early diagnosis. With a timely recognition of the catastrophe there is some hope that surgical treatment will save the patient's life; without it there is practically none. Operation must be prompt if it is to be effectual.

With regard to the relative frequency of perforation in typhoid fever, statistics show that of the cases treated in the London hospitals during the last decade more than one-fourth of all the deaths from enteric fever were from this cause, and more than 3 per cent. of all who are attacked by the disease lose their lives in this way. The accident is one which it is impossible to guard against or foresee; it occurs in the mildest cases as well as the most severe. The danger is present throughout the whole of the illness. It commences with the onset, and is not over until complete recovery has taken place. The greatest risk, however, is during the second, third, and fourth weeks.

Of the early symptoms of perforation, abdominal pain is by far the most important. The pain usually comes on suddenly, is probably severe and persistent, and is most commonly referred to the lower half of the abdomen. Vomiting, nausea, hiccough, action of the bowels repeated at short intervals, a rigor, sweating, mental excitement, or the symptoms of collapse, may any of them precede, accompany, or quickly follow the onset of pain. Confirmatory signs will be: (1) A change in the temperature; either a sudden drop or a sudden rise. (2) Increased frequency and diminished force of the pulse. (3) The development of a pinched, drawn expression. Still more significant than these are the local signs found on examination of the abdomen. These are: (1) Tenderness of the abdomen on palpation, often general but usually more marked over the lower half. (2) Rigidity of the abdominal walls. (3) Obliteration of the liver-dulness. (4) Shifting dulness in the flanks. (5) Diminished movement of the abdomen with respiration. If it be found that the abdominal wall is flaccid and soft and moves freely with respiration, that the liver-dulness is normal in extent, and that the flanks are resonant, then one may rest assured that as yet no perforation has occurred. Leucocytosis is an almost constant result of perforation, but may equally result from other complications, so that as a diagnostic aid its absence is of greater significance than its presence.

[1] Practitioner, January, 1904.

